# Fenofibrate monotherapy-induced rhabdomyolysis in a patient with post-pancreatitis diabetes mellitus: A rare case report and a review of the literature

**DOI:** 10.1097/MD.0000000000020390

**Published:** 2020-05-22

**Authors:** Jingjing Zhou, Dongfeng Li, Qiansong Cheng

**Affiliations:** a Department of Endocrinology of Lu’an Second People's Hospital, The Affiliated Hospital of West Anhui Health Vocational College; b Department of Hematology of Lu’an People's Hospital, The Lu’an Affiliated Hospital of Anhui Medical University, Lu’an, Anhui, China.

**Keywords:** acute renal failure, diabetes mellitus, fenofibrate, pancreatitis, rhabdomyolysis

## Abstract

**Rationale:**

Fibrates are widely used to control hypertriglyceridemia and mixed dyslipidemia alone or in combination with statins. These drugs have rare, but severe and potentially vital adverse reactions of rhabdomyolysis and secondary acute renal failure (ARF). The objective of this article is to analyze this adverse effect of fibrates and ensure the safety of drug use.

**Patient concerns:**

We report a case of rhabdomyolysis and ARF due to fenofibrate monotherapy in a 68-year-old female with post-pancreatitis diabetes mellitus and review reported cases of rhabdomyolysis correlated with fibrates monotherapy.

**Diagnosis:**

The patient was diagnosed with rhabdomyolysis associated with fenofibrate monotherapy as confirmed by symptoms of fatigue and muscle pain, and elevated levels of myoglobin and creatine kinase.

**Interventions:**

Fenofibrate therapy was discontinued. Moreover, intravenous fluids, urinary alkalization, and diuretic were performed.

**Outcomes:**

The symptoms were completely relieved, and relevant laboratory indexes returned to normal range during follow-up.

**Lessons:**

Physicians should be aware of the side effect of rhabdomyolysis of fibrates, and patients should also be informed about this potential side effect, especially for patients with high-risk factors. A favorable outcome can be achieved by timely diagnosis and prompt treatment.

## 1 Introduction

Fibrates are widely used as effective drugs in the treatment of diabetic hypertriglyceridemia and mixed dyslipidemia alone or in combination with statins.^[1]^ Frequent side effects of fibrates are gastrointestinal discomfort, musculoskeletal symptoms, vertigo, headache, anxiety, skin rash, and loss of libido.^[2,3]^ Rhabdomyolysis is often associated with myoglobinuria and acute renal failure (ARF), which is a rare but serious adverse event of fibrates monotherapy. The risk of rhabdomyolysis with fenofibrate monotherapy increases in patients with hypothyroidism, diabetes mellitus, renal disease, female gender, and/or older age.^[4]^ Here, we report a novel case of rhabdomyolysis induced by fenofibrate monotherapy in a patient with post-pancreatitis diabetes, and review the reported cases of rhabdomyolysis associated with fibrates monotherapy. Informed consent was obtained from the patient for publication of the case.

## 2 Case report

A 68-year-old female patient was admitted to our hospital with complaints of fatigue, muscle pain, and a decreased urine output for the previous 2 weeks. She had a history of diabetes mellitus after acute pancreatitis for nearly 11 years, accompanied by peripheral vascular disease and peripheral neuropathy. Moreover, she had suffered from dyslipidemia for about 2 years. Her regular medications included insulin (totaling 16 units daily) and mecobalamin (3 tablets daily). 250 mg micronized fenofibrate daily was initiated to control hypertriglyceridemia 5 months ago. She had no history of liver, muscle, or kidney disease, and had not caught a cold recently. She also denied hypertension, coronary heart disease, or thyroid disease. Diffuse tenderness of muscles and pretibial oedema were detected on physical examination. No obvious abnormality was found in chest X-ray, abdominal ultrasonography, and cardiac ultrasonography. The electrocardiogram showed sinus rhythm without alteration of ST segment. On the basis of the above data, there was no evidence of acute myocardial infarction.

The results of the laboratory examination on admission were as follows: creatine kinase (CK): 8385 IU/L (normal: 24–174 IU/L), aspartate aminotransferase: 716 IU/L (normal: 0–40 IU/L), alanine aminotransferase: 68 IU/L (normal: 0–40 IU/L), lactate dehydrogenase: 1244 IU/L (normal: 103–227 U/L), serum creatinine: 97.63 μmol/L (normal: 41–81 μmol/L), blood urea nitrogen: 14.79 mmol/L (normal: 1.7–8.3 mmol/L), myoglobin: 1075 ng/mL (normal: 25–72 ng/mL), troponin T: 37.28 ng/L (normal: 0–14 ng/L). Antinuclear antibody, anti-JO-1 antibody, anti-double-stranded DNA antibody, anti-centromere antibody, anti-mitochondrial antibody M2, and rheumatoid factor were all negative. Thyroid stimulating hormone (TSH), total thyroxin 3 (TT3), and total thyroxin 4 (TT4) were normal. No significant abnormality was found in other laboratory tests. Therefore, a diagnosis of rhabdomyolysis and secondary ARF due to fenofibrate monotherapy was made.

Because of the serious side effect, fenofibrate was discontinued. Fluid infusion, urine alkalization with sodium bicarbonate, diuretic with torasemide, and hepatoprotection were performed subsequently. The patient's complaints were ameliorated and urine output increased gradually during the hospitalization. On the fifth day of treatment, serum urea nitrogen and creatinine returned to normal range. Although the patient's CK concentration decreased to 2184.8 U/L, which did not return to baseline level, she was discharged 6 days after hospitalization and monitored as an outpatient. Two weeks after discharge, the patient's symptoms disappeared, and serum CK, creatinine, and myoglobin were completely normal (Table 1).

**Table 1:**
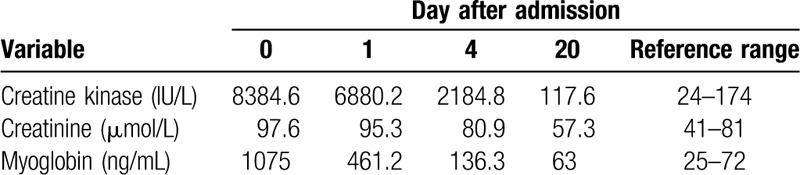
Relevant laboratory values of our patient after the admission.

## 3 Discussion

Rhabdomyolysis or myopathy can occur after diverse types of acute muscle insults. Damaged muscle releases intracellular substances into the systemic circulation as toxins, especially in the kidney. Patients with rhabdomyolysis have symptoms characterized by diffuse muscle pain, fatigue, weakness, and elevated serum levels of CK. Rhabdomyolysis is defined as serum CK concentration 10 times higher than the upper limit of normal.^[5]^ This disorder has been found to be correlated with the use of lipid-lowering drugs, including statins and fibrates, especially when used in combination.^[6]^ Compared with statins, fibrates are generally well tolerated. Frequent side effects of fibrates are gastrointestinal discomfort, musculoskeletal symptoms, vertigo, headache, anxiety, skin rash, and loss of libido. Rhabdomyolysis is a rare, but severe and potentially virulent adverse reaction with fibrates therapy.

Up to date, only a few cases of rhabdomyolysis induced by fibrates monotherapy have been reported. Here, we reported a novel case of fenofibrate monotherapy-induced rhabdomyolysis in a patient with post-pancreatitis diabetes mellitus (DM). The present report depicted a diabetic patient with only a history of hypertriglyceridemia and acute pancreatitis, without other past medical history and medication history. The patient was diagnosed with rhabdomyolysis on the basis of fatigue, muscle pain, oliguria, marked increases in CK, and myoglobin. After discontinuation of fenofibrate, hydration, alkalization, and diuresis, the patient was cured. Due to the severe side effect, the patient did not resume lipid-lowering therapy with fenofibrate or other hypolipidemic drugs during follow-up. Through diet and exercise, the blood lipid control of the patient was poor and triglyceride gradually increased (data not shown). Therefore, for patients with fenofibrate-induced rhabdomyolysis, how to choose appropriate lipid-lowering drugs is a challenge, which needs further research.

Until now, the definite association between fibrates and rhabdomyolysis remains unclear. Fibrates may affect cholesterol biosynthesis and lead to changes in the constitution and function of the muscle fibre's plasma membrane.^[7]^ Emergence of muscle necrosis and increase in muscular lipoproteinlipase activity may also be the underlying mechanisms of fibrates-induced rhabdomyolysis.^[7]^ It is demonstrated that fibrates can induce specific cell injury to human embryonal rhabdomyosarcoma cells through activation of peroxisome proliferator-activated receptor-α (PPARα).^[8]^ Therefore, activation of PPARα-signaling pathway may be an important mechanism of fibrates-induced rhabdomyolysis.

Fenofibrate monotherapy-induced, and diabetes-related rhabdomyolysis were rarely reported, with only 5 cases having been described in English literature.^[9–13]^ We present an additional case, which is the first report of rhabdomyolysis induced by fenofibrate monotherapy in a patient with post-pancreatitis DM. DM is associated with decreased mitochondrial and stem cell functions in skeletal muscle cells. These dysfunctions can affect cell homeostasis and lead to muscle disorders in diabetic patients.^[14]^ These abnormalities may also make muscle cells susceptible to other factors causing muscle damage and increase the risk of rhabdomyolysis. The level of circulating pro-inflammatory cytokines, including IL-6, TNF-a, and monocyte chemoattractant protein (MCP)-1, increases significantly in patients after acute pancreatitis.^[15]^ Circulating IL-6 may participate in mitochondrial DNA damage and iron dyshomeostasis of skeletal muscle cells, which is implicated in muscle aging. This maybe one of the potential risk factors for rhabdomyolysis.^[16]^ Due to the scarcity of cases, the exact mechanism of fenofibrate-induced rhabdomyolysis in diabetic patients, especially in post-pancreatitis DM patients, remains unclear and needs further study.

For an in-depth study and a preferable understanding of patient characteristic, risk factor, clinical management, and prognosis of this adverse effect, we made further efforts to search for studies of rhabdomyolysis induced by fibrates monotherapy in the PubMed database. The relevant studies reported in the English-language literature were searched from onset to August 20, 2019 using the following terms: fibrates, gemfibrozil, fenofibrate, ciprofibrate, bezafibrate, and rhabdomyolysis. Due to severe adverse reactions of clofibrate and rare utilization of it at present, the cases of rhabdomyolysis associated with clofibrate were excluded. Moreover, the references of all included articles were manually retrieved to ensure that no cases were lost. The cases were excluded if they fulfilled the following criteria:

1. no detailed patient, clinical, and laboratory records, and/or
2. used in combination with statins or other drugs that had a causal relationship with rhabdomyolysis.

A total of 31 cases of rhabdomyolysis induced by fibrates monotherapy were found, including 16 cases of fenofibrate, ^[4,5,9–13,17–25]^ 8 cases of bezafibrate,^[26–32]^ 5 cases of gemfibrozil,^[33–37]^ and 2 cases of ciprofibrate.^[38,39]^ All clinical and laboratory data were summarized in Table 2. There was no apparent gender difference in fibrates monotherapy-induced rhabdomyolysis. Rhabdomyolysis frequently occurred in people with advanced age, hypertension, DM, chronic renal failure, and/or hypothyroidism. Therefore, these may be potential risk factors for rhabdomyolysis associated with fibrates monotherapy. Time of reaction initiation varied between 3 days and 3 years (the average time was 17.5 weeks). Most patients with rhabdomyolysis were complicated with secondary ARF. Most sufferers can be cured by discontinuation of fibrates, or in combination with hydration, alkalinization, and diuresis. Other strategies were needed for few cases with severe renal insufficiency, including hemodialysis, continuous renal replacement therapy (CRRT), and plasma exchange. The prognosis of rhabdomyolysis was well, and most patients can recover and improve.

**Table 2:**
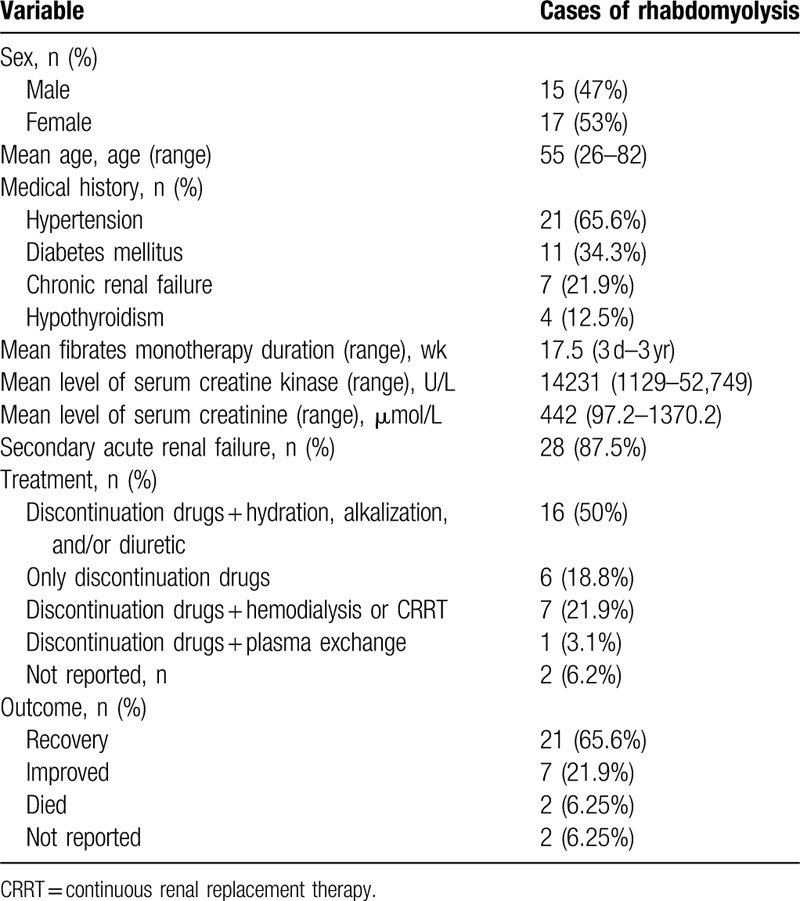
Summary of 31 previously reported cases (30 articles) of rhabdomyolysis associated with fibrates monotherapy and the present case.

## 4 Conclusion

We report a novel case of fenofibrate monotherapy-induced rhabdomyolysis in a patient with post-pancreatitis DM, and literature review as well as our case observation suggests that prescribers and users of fibrates should be mindful to its rare, but potentially fatal adverse effects such as rhabdomyolysis and secondary ARF. For patients taking fibrates, risk factors such as old age, hypertension, DM, chronic renal failure, and hypothyroidism should be regarded. Satisfactory results can be achieved by timely diagnosis and effective treatment for patients with fibrates-induced rhabdomyolysis.

## Author contributions

**Conceptualization:** Jingjing Zhou.

**Data curation:** Jingjing Zhou, Dongfeng Li.

**Supervision:** Dongfeng Li, Qiansong Cheng.

**Writing – original draft:** Jingjing Zhou.

**Writing – review & editing:** Jingjing Zhou, Qiansong Cheng.
